# Gaps in the implementation of national core elements for sustainable antimicrobial use in the WHO-African region

**DOI:** 10.3389/frabi.2022.1047565

**Published:** 2022-12-02

**Authors:** Walter L. Fuller, Aaron O. Aboderin, Ali Yahaya, Adeyemi T. Adeyemo, Laetitia Gahimbare, Otridah Kapona, Omotayo T. Hamzat, Oumar Bassoum

**Affiliations:** ^1^ Assistant Regional Director Cluster, World Health Organization Regional Office for Africa, Brazzaville, Republic of Congo; ^2^ Department of Medical Microbiology, College of Health Sciences, Obafemi Awolowo University, Ile-Ife, Nigeria; ^3^ Department of Medical Microbiology, Obafemi Awolowo University Teaching Hospitals Complex, Ile-Ife, Nigeria; ^4^ Zambia National Public Health Institute, Lusaka, Zambia; ^5^ Laboratory Systems and Network, Zambia National Public Health Institute, Lusaka, Zambia; ^6^ Faculté de Médecine, de Pharmacie et d’Odontologie, Université Cheikh Anta Diop de Dakar, Dakar-Fann, Senegal

**Keywords:** antimicrobial stewardship, national core elements, WHO-AFRO, AWaRe classification, AMS programs, survey

## Abstract

**Background:**

Antimicrobial resistance (AMR) has emerged as a leading global health and economic threat of the 21st century, with Africa bearing the greatest burden of mortality from drug-resistant infections. Optimization of the use of antimicrobials is a core strategic element of the response to AMR, addressing misuse and overuse as primary drivers. Effectively, this requires the whole society comprising not only healthcare professionals but also the public, as well as the government, to engage in a bottom-up and a top-down approach. We determined the progress of African national governments in optimizing antimicrobial drug use.

**Methods:**

From September 2021 to June 2022, all 47 member states of the World Health Organization African region (WHO AFRO) were invited to participate in a survey determining the implementation of strategies to optimize antimicrobial use (AMU). We used the WHO antimicrobial stewardship (AMS) assessment tool, *National core elements—A checklist to guide the country in identifying existing national core elements for the implementation of AMS Programs*, to obtain information from national AMR focal persons. The tool consists of four sections—national plans and strategies; regulations and guidelines; awareness, training, and education; and supporting technologies and data—with a total of 33 checklist items, each graded from 0 to 4. The responses were aggregated and analyzed using Microsoft Excel 2020^®^.

**Results:**

Thirty-one (66%) of the 47 countries returned completed forms. Only eight (25.8%) countries have developed a national AMS implementation policy incorporating defined goals, targets, and operational plans. There are no budget lines for AMS activities in 23 (74.2%) countries. The WHO Access, Watch, Reserve (AWaRe) classification of optimizing AMU has been integrated into the national essential medicines list or formulary in 19 (61.3%) countries, while the incorporation of the AMS principles and WHO AWaRe classification into national clinical guidelines for the management of infections is present in only 12 (38.7%) and 11 (34.5%) countries, respectively. Although regulations on the prescription-only sale/dispensing of antibiotics are present in 68% of countries, their enforcement is poor. Systems identifying pathogens and antibiotic susceptibility for optimal use of antibiotics are lacking in 38% of countries.

**Conclusion:**

In Africa, wide gaps exist in the governments’ implementation of the core elements of optimizing antimicrobial drug use. Responding to AMR constitutes a long journey, and technical and financial support needs to be deployed to optimize the use of antimicrobials.

## Background

Antimicrobial resistance (AMR) poses an enormous threat to the sustainability of human, animal, and plant health ecosystems, with huge burdens of morbidity, mortality, and healthcare costs. It is a consequential global public health problem with far-reaching implications on the global economy and prosperity and on the overall development of states and nations (Majumder et al., 2020). AMR is a cross-cutting interdisciplinary issue associated with significant deleterious impacts on human health, food and environmental security, and the achievement of Sustainable Development Goals (SDGs) (World Health Organization, 2017). Africa, with its inherent heavy burden of communicable and non-communicable diseases, poverty, inadequate health systems and infrastructure, and poor governance and corruption, bears a huge brunt of the global AMR burden (Jasovský et al., 2016; Tadesse et al., 2017). At the current rates, AMR will cause an estimated 10 million deaths every year, leading to 2%–3.5% reductions in productivity by 2050 (Antimicrobial Resistance Collaborators, 2022). Globally, an estimated 4.95 million deaths were associated with bacterial AMR in 2019, with the death burden highest in western Sub-Saharan Africa at 27.3 per 100,000 (Antimicrobial Resistance Collaborators, 2022). The primary driver of AMR is overuse, with the misuse of antimicrobials in humans, animals, and plants more pronounced in low- and middle-income countries (LMICs) as a result of uncontrolled access, inappropriate and excessive prescribing by healthcare practitioners, and the lack or non-utilization of treatment guidelines in case management (Engler et al., 2021). The overuse and the misuse of antimicrobials are pervasive across countries in Africa, which are results of poor regulations, lack of awareness and training, poor access to quality-assured antimicrobial medicines, insufficient diagnostic microbiology capacities, and socioeconomic and cultural limitations (Alhaji and Isola, 2018; Akpana et al., 2020). A more recent significant driver of AMR is the coronavirus disease 2019 (COVID-19) pandemic, as shown by a large meta-analysis, in which the prevalence of antibiotic prescribing was 74.6% compared to the estimated bacterial co-infection of 8.6% in patients with COVID-19 (Langford et al., 2021).

Global antibiotic consumption increased markedly by 65% from 2000 to 2015, with LMICs contributing the largest proportion (Klein et al., 2017). The global approach to addressing the threat of AMR, including the World Health Organization’s (WHO) global action plan on AMR, advocates antimicrobial stewardship (AMS) as a core strategy for optimizing the use of antimicrobial medicines (World Health Organization, 2015).

The WHO advocates for AMS programs across continents and, in 2019, developed a guidance toolkit to enable the implementation of AMS programs in LMICs, detailing the core elements of AMS at both national and healthcare facility levels (World Health Organization, 2019). There is a paucity of published data on the implementation of AMS programs in Africa. A systematic review of the literature in five electronic databases covering over 30 years (1990–2019) could only identify 13 studies that met the defined inclusion criteria, with all the studies limited to only five countries, namely, South Africa, Kenya, Sudan, Tanzania, and Egypt (Akpana et al., 2020). A different systematic review of the literature without restrictions on the date of publications until 2020 in Embase and Ovid MEDLINER^®^ databases determined barriers and facilitators of AMS programs and identified 14 eligible studies from 11 African countries. Many of the studies, rather than being nationally representative, were confined to regions within the countries (Porter et al., 2021). A more recent scoping review of the AMS landscape in eight (Ghana, Nigeria, Sierra Leone, Kenya, Tanzania, Uganda, Malawi, and Zambia) countries in Africa revealed variations in the implementation of AMS programs, with only Kenya having national AMS guidelines for healthcare settings (Kamere et al., 2022). The Kenyan guidelines provide direction for actors in the implementation of AMS at different service centers including hospitals, outpatient clinics, and community pharmacies.

We sought to determine the Africa-wide status of the implementation of AMS programs following the support of the quadripartite and other partners in the implementation of national action plans (NAPs) on AMR in Africa (Fuller et al., 2022). Our survey assessed the national-level implementation of AMS programs in 31 out of the 47 countries within the WHO African region (WHO AFRO), with a view to informing appropriate interventions for improvement.

## Materials and methods

### Assessment tool

We used the WHO’s *National core elements: A checklist to guide the country in identifying existing national core elements for the implementation of AMS Programs* (World Health Organization, 2021). Briefly, the checklist includes an introductory *General information* section that documents information about the country, the respondents, and the person administering the questionnaire. The next section on *National core elements of AMS program* has four domains: *Core element 1—National plans and strategies* (11 items); *Core element 2—Regulations and guidelines* (13 items); *Core element 3—Awareness, training, and education* (7 items); and *Core element 4—Supporting technologies and data* (2 items). Appropriateness of the responses to the checklist is established by means of verifiers tagged to each of the items. Each of the total 33 checklist items is scored from 0 to 4, as follows:

0 = No, the core element is not in place and is not a priority;
1 = No, the core element is a priority, but there is no plan in place to initiate it;
2 = The core element is planned, but no action has taken place;
3 = The core element is in place, but it is only partially implemented, requiring further strengthening; and
4 = The core element is in place and is fully implemented without requiring strengthening, but needing to be sustained.

The total score on the checklist for each country was determined by adding the scores for each of the 33 items on the checklist, with a range of 33–132. The total score obtained was used to determine the assigned AMS core element level. There are four possible AMS core element levels: inadequate (score of 0–33), basic (score of 34–66), intermediate (score of 67–99), and advanced (score of 100–132).

### Respondents

The survey tool, as designed by the WHO, was piloted in five member states in WHO AFRO between September and December 2021. Between June and September 2022, without further modifications, the survey questionnaire was e-mailed by the Antimicrobial Stewardship and Awareness Unit at the WHO AFRO headquarters, Brazzaville, Congo, to AMR focal persons in the member states for onward transmission to respective national AMR focal persons in the Ministries of Health or National Public Health Institutes, who coordinated and returned completed questionnaires. For each of the member states, one duly completed form was filled out and returned.

### Data analysis

Responses were received as Microsoft Word files. The data were aggregated and analyzed using Microsoft Excel 2020 (Redmond, WA, USA).

## Results

**Figure 1:**
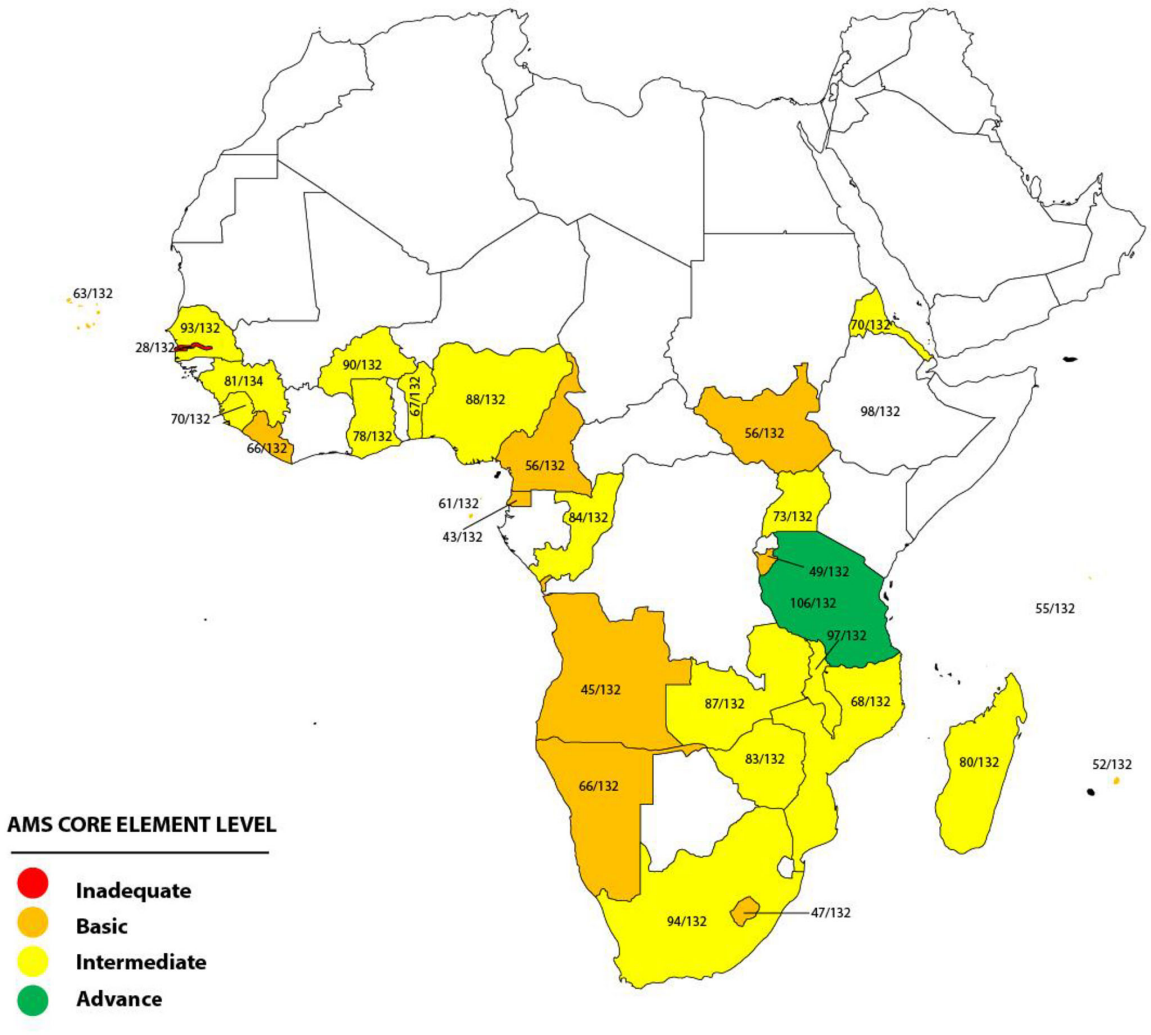
Antimicrobial stewardship (AMS) core element levels.

A total of 31 (66%) out of the 47 WHO AFRO member states, which are distributed across the Central, East, West, and Southern African regions, returned the duly completed assessment tool by September 2022. The survey assessed the implementation of the core elements of the AMS program at the national government level in the 31 African countries and categorized each country into one of four possible AMS core element levels based on the total scores on the assessment checklist. There were 12 countries that scored 34–66, categorized as *basic AMS core element level*, while 17 countries scored 67–77, included in the category *intermediate AMS core element level*. One of the remaining two countries scored 106 (*advanced AMS core element level*), while the other scored 28 (*inadequate AMS core element level*) (**Figure 1**). For each of the four domains on the assessment checklist of national core elements, regulations and guidelines had the highest level of implementation, while awareness, training, and education had the least.

### National plans and strategies

Assessment of the 11 checklist items that constitute national plans and strategies showed that there were no NAPs on AMR that prioritize AMS or national multisectoral coordinating committees on AMR in nine (29.0%) and eight (25.8%) countries, respectively. Dedicated funding either by streamlining into the existing domestic budget line or through external funding is unavailable for NAPs on AMR and national AMS activities in 23 (74.2%) and 25 (80.6%) countries, respectively. National structures providing governance and coordination for AMS, including AMS technical working groups (TWGs) with clear terms of reference (TOR), are not in place in 18 (58.1%) countries, while only in 10 countries do the national AMS TWGs schedule meetings, generate reports, and also report to the National AMR Committee. Linkages between AMS TWGs and existing TWGs, such as infection prevention and control (IPC), exist in eight (25.8%) countries. AMS implementation plans or policies with defined goals and timelines that are endorsed at the national level are only available in eight (25.8%) countries, with the same number linking to other national plans, such as IPC and WASH (water, sanitation, and hygiene) (**Table 1**).

**Table 1:** Core element 1—national plans and strategies (frequencies of scores).

Assessment parameters	0 = No, the core element is not in place and is not a priority	1 = No, the core element is a priority, but there is no plan in place to initiate it	2 = The core element is planned, but no action has taken place	3 = The core element is in place, but it is only partially implemented, requiring further strengthening	4 = The core element is in place and is fully implemented without requiring strengthening, but needing to be sustained
	No. of countries with the score categories				
Presence of NAPs on AMR prioritizing AMS	0	2	7	11	11
Presence of a multi-sectoral coordinating committee on AMR	0	4	4	7	16
Dedicated funding for NAPs on AMR	9	11	3	8	0
Dedicated funding for AMS activities in the NAPs on AMR	7	10	8	5	1
Presence of AMS TWGs with clear TOR	3	9	6	8	5
Linkage of AMS TWGs to other TWGs	7	6	10	5	3
AMS TWGs meeting on a regular basis	8	4	9	4	6
AMS TWGs reporting back to a national AMR coordinating committee	8	5	8	6	4
Presence of a national AMS implementation policy with defined goals and targets and operational plans	7	9	7	4	4
Linkage of a national AMS implementation plan to other plans	9	10	6	4	2
Presence of mechanisms to monitor and evaluate progress on implementing the NAPs on AMR	5	4	11	8	3

NAPs, national action plans; AMR, antimicrobial resistance; AMS, antimicrobial stewardship; TWGs, technical working groups; TOR, terms of reference.

### Regulations and guidelines

All of the countries, with the exception of only two, have a national essential medicines list (EML) or formulary to guide the prescription of drugs. However, only 19 (61.3%) countries have integrated the WHO Access, Watch, Reserve (AWaRe) classification into the national EML or formulary. Up-to-date clinical guidelines for the management of infections are available in 20 (64.5%) countries, but the incorporation of AMS principles and the WHO AWaRe classification into the guidelines is present in only 12 (38.7%) and 11 (34.5%) countries, respectively. In countries where there are no guidelines present, only two indicated the availability of human and financial resources to support the development of such clinical guidelines.

Regulations that ban fixed-dose combinations of antibiotics not recommended in international or national treatment guidelines are available only in three (9.7%) countries. On the other hand, regulations on the prescription-only sale/dispensing of antibiotics are common, and are present in most (21/31) of the countries, although only 14 countries enforce such regulations.

A national medicines authority/agency that ensures the availability of quality antibiotics exists in 27 (87.1%) countries, although reporting mechanisms for shortages or stockouts and substandard and falsified antibiotics are available only in 19 (61.3%) and 21 (67.7%) countries, respectively (**Table 2**).

**Table 2:** Core element 2—regulations and guidelines (frequencies of scores).

Assessment parameters	0 = No, the core element is not in place and is not a priority	1 = No, the core element is a priority, but there is no plan in place to initiate it	2 = The core element is planned, but no action has taken place	3 = The core element is in place, but it is only partially implemented, requiring further strengthening	4 = The core element is in place and is fully implemented without requiring strengthening, but needing to be sustained
	No. of countries with the score categories				
Availability of a national essential medicines list or formulary to guide prescribing	1	1	0	7	22
Integration of the AWaRe classification into the national EML or formulary	2	7	3	3	16
Availability of up-to-date clinical guidelines for the management of infections	3	6	2	9	11
Clinical guidelines incorporating AMS principles	7	8	4	7	5
Clinical guidelines integrating AWaRe classification	6	10	4	3	8
Availability of human and financial resources to support the development of clinical guidelines	7	9	6	5	4
Availability of regulations that ban fixed-dose antibiotic combinations	13	12	3	1	2
Presence of regulations on the prescription-only sale/dispensing of antibiotics	2	5	3	6	15
Enforcement of regulations on dispensing antibiotics by prescription only	4	10	3	11	3
Availability of a national medicines authority that ensures the availability of quality antibiotics	0	3	1	3	24
Presence of mechanisms for reporting shortages and stockouts of antibiotics in the country	2	8	2	10	9
Presence of mechanisms in place to report substandard or falsified antibiotics/antimicrobials in the country	1	7	2	10	11
Presence of a relevant agency ensuring the availability and affordability of antibiotics in suitable dosage forms	0	1	1	14	15

AMS, antimicrobial stewardship.

### Core element 3—Awareness, training, and education

Most countries (23/31) in the region hold regular (at least annually) public awareness campaigns on AMR and rational antibiotic use; contrariwise, evidence for basic knowledge in schools/students of the rational use of antibiotics and IPC is provided only in 8 (25.8%) countries. Varying levels of in-service training on antimicrobial prescribing that target AMS teams are provided by the government only in 11 (35.5%) countries, while those that target general healthcare professionals are offered in 14 out of 31 (45.2%) countries. In the same vein, only 45.2% of countries have their educational curricula incorporating AMS for healthcare professionals. Government incentives to support the implementation of AMS programs in healthcare facilities, including staffing standards, training, and accreditation, are almost not in existence (3/31); neither are there criteria set for AMS programs in the accreditation of healthcare facilities in all but one (3.2%) country (**Table 3**).

**Table 3:** Core element 3—awareness, training, and education (frequencies of scores).

Assessment parameters	0 = No, the core element is not in place and is not a priority	1 = No, the core element is a priority, but there is no plan in place to initiate it	2 = The core element is planned, but no action has taken place	3 = The core element is in place, but it is only partially implemented, requiring further strengthening	4 = The core element is in place and is fully implemented without requiring strengthening, but needing to be sustained
	No. of countries with the score categories				
Regular public awareness campaigns on AMR and the responsible/rational use of antibiotics at the country level	3	3	2	13	10
Government providing education on IPC and rational use of antibiotics in schools at basic, primary, and secondary levels	4	11	8	7	1
Government facilitating access to in-service training on antimicrobial prescribing and stewardship for AMS teams	1	11	8	11	0
Government facilitating access to in-service training and CPD on antimicrobial prescribing and AMS for healthcare professionals	2	6	9	10	4
Inclusion of AMS principles and strategies in the educational curriculum of healthcare professionals	0	8	9	12	2
Government supporting the implementation of AMS programs in all healthcare facilities	11	11	6	3	0
Government set the criteria for AMS programs in healthcare facilities for accreditation in the country	13	10	7	0	1

AMR, antimicrobial resistance; IPC, infection prevention and control; AMS, antimicrobial stewardship; CPD, continuing professional development.

### Core element 4: Supporting technologies and data

A total of 61% (19/31) of the countries have systems in place for pathogen identification and antibiotic susceptibility testing to guide optimal antibiotic use for direct patient care, as well as updating available treatment guidelines, and less than a third (10/31, 32.3%) of the countries have systems in place to collect, analyze, and disseminate national antimicrobial consumption (AMC) surveillance data (**Table 4**).

**Table 4:** Core element 4—supporting technologies and data.

Countries	System in place to collect, analyze, and disseminate national antimicrobial consumption surveillance data	System in place to identify pathogens and antibiotic susceptibility to guide optimal use of antibiotics over time in clinical practice and update clinical guidelines
	Scores	
Angola	1	2
Benin	3	3
Burkina Faso	3	3
Burundi	2	3
Cabo Verde	0	3
Cameroon	2	3
DR Congo	2	1
Eritrea	1	2
Ethiopia	3	3
Gambia	0	3
Ghana	2	2
Equatorial Guinea	2	2
Guinea	1	4
Lesotho	0	2
Liberia	1	1
Madagascar	1	3
Malawi	3	4
Mauritius	1	3
Mozambique	3	3
Namibia	2	2
Nigeria	2	1
São Tomé	0	0
Senegal	2	3
Seychelles	1	3
Sierra Leone	1	1
South Africa	4	3
South Sudan	1	1
Tanzania	3	3
Uganda	4	4
Zambia	4	4
Zimbabwe	3	3

## Discussion

AMR is a silent pandemic, identified as one of the top 10 global public threats (E Clinical Medicine, 2021), and a global priority to mitigate. The enormity of AMR is far more pronounced in Africa, partly attributable to the high burden of infectious diseases complicated by poor IPC practices fueling excessive AMC and the emergence of AMR (Kakkar et al., 2020). In Africa and many other LMICs, there are striking impediments to AMR control, including the lack of political commitments and poor governance, which result in inadequate regulations and poor health financing (Pokharel et al., 2019). While AMS programs are successful in many developed countries (Huttner et al., 2014), such programs are just evolving with enormous implementation challenges in many LMICs (Cox et al., 2017). There is a dearth of information on the country-level progress of ASP within the African continent, and only a few available data revealed that AMS has gained more ground in South and East Africa compared to West Africa. A systematic review of the implementation of AMS programs in Africa included studies primarily from South Africa and a few East African countries, with none of the 13 studies from West Africa (Akpana et al., 2020). A review of 14 Sub-Saharan Africa studies clearly elucidated that the barriers to AMS included the lack of regulations or their enforcement on the prescriptions and sales of antimicrobials at the country or the regional level, the heterogeneity and complex nature of the healthcare system, poor clinical governance typified by the lack of AMS guidelines and poor adherence to such guidelines, when available, and the lack of national support for AMS in terms of human and financial resources and laboratory facilities (Porter et al., 2021). In these developing countries, there are action plans for AMS at the national or sub-national level; however, in principle, implementation is often hindered by low political commitment and the lack of operational policies, as well as legal and regulatory frameworks, with escalated effects on the development of facility-based systems (Kakkar et al., 2020).

### Dedicated funding for AMS activities

Only 6 out of the 31 countries assessed have budgetary provisions and dedicated funds to support national AMS programs. This is in contrast to high-income countries where national governments provide such funding, for example, in England through the Department of Health guidelines and hospital inspection. This is similar to Norway and France, where government-led health indicators facilitate the development of AMS programs in hospitals. Although perceived as essential, state support is lacking in many LMICs, constituting a key barrier to implementing and developing AMS programs (Gebretekle et al., 2018; Charani et al., 2019).

Notwithstanding the poor local and national support in resource-limited countries for curtailing AMR utilizing multifaceted approaches, including AMS programs, there are documented efforts and support from global partners. Different members of the quadripartite, including the WHO, Food and Agricultural Organization, the World Organization for Animal Health, and other multilateral organizations, support national governments and collaborate with other partners to upscale efforts at controlling AMR in key areas such as awareness, education, and training; national surveillance; policy and practice; and multi-sectoral collaboration and coordination (WHO, 2019).

Beyond funding and increasing international efforts for AMS activities (Centre for Infectious Disease Research and Policy, 2021; Tattevin et al., 2020), countries within the region need to link AMR to other national programs and strategies in order to ensure sustainable support and progress for investment and accountability.

### AMS implementation policies and plans

Only 8 (25.8%) out of the 31 countries have national AMS implementation plans or policies with defined goals, targets, and operational plans, which is a reflection of the country-level efforts prioritizing AMS as a core element for addressing the threat of AMR being low. This finding of a lack of AMS blueprint mirrors what has been reported earlier that only one out of eight countries has national AMS guidelines to impact practices (Kamere et al., 2022). In settings where some AMS policies are available, practical implementation is mostly challenged by financial, structural, and behavioral barriers, as well as competing national priorities. There have been working initiatives across Africa to improve AMS utilizing collaborative programs and partnerships, such as the African Institute for Development Policy (AFIDEP) in Malawi, Uganda and Malawi’s Drivers of Resistance in Uganda and Malawi (DRUM), the ReAct Africa powered experts’ and stakeholders’ meetings to form technical working groups on AMR in countries such as Zambia and Ghana, and the country-driven antimicrobial use (AMU) awareness efforts in Nigeria (Kamere et al., 2022).

The feedback from countries elucidated the need to upscale the availability of implementable policies and plans in Africa to drive AMS programs and the importance of paying attention to the implementation of such policies and plans by giving priority to mobilizing necessary machinery within countries and leveraging on initiatives that incorporate local and foreign partners for continent-wide progress.

### Enforcement of regulations guiding access to antimicrobials

A lot of African countries have regulations in place for the prescription-only sale/dispensing of antimicrobials and authorities and mechanisms for ensuring quality and reporting substandard or falsified drugs; however, the enforcement of these laws and regulations is largely poor. The present study shows obvious gaps in the enforcement of country regulations for the prescription-only dispensing of antibiotics, signifying the widespread availability of over-the-counter antibiotics. Ineffective national regulations and poorly functioning regulatory strategies are common issues in Africa, immensely contributing to the uncontrolled availability and use of antibiotics across the continent (Akpana et al., 2020). A systematic review of the literature across Sub-Saharan Africa highlighted the deficiencies in the antimicrobial regulations at the country or regional level (Porter et al., 2021). A report documented Ghana having available laws that control the use of antimicrobials in humans, but the enforcement of these laws is weak, leading to the supply of antibiotics to and from unauthorized outlets (Yevutsey et al., 2017). In Tanzania, for example, notwithstanding the strict regulatory policy to ensure that antibiotics are prescription-only medicines and sold under strict control, irrational and excessive utilization is commonplace (Dhingra et al., 2020). Likewise, in many other countries including Nigeria, Mozambique, Ethiopia, and Burkina Faso, the laws and regulations are enacted governing the use of medicine with relevant regulatory agencies and framework, but the drive and will to enforce such laws are also weak or totally lacking (Ndihokubwayo et al., 2013; Huttner et al., 2014; Egwuenu et al., 2018). The unconscionable situation of drug regulations in WHO AFRO calls for strengthened national drug regulatory systems through concerted efforts engaging all the key stakeholders. Beyond the need for an improved commitment to enforcing extant laws on the path of individual countries, there should be effective and operational regional and interregional collaboration and synergy to reinforce the existing regulatory networks and capacities in the region, leveraging on advances in digital technology without compromising access to essential antimicrobials.

### Availability and adherence to clinical guidelines

A total of 35% of the countries do not have up-to-date antibiotic treatment guidelines; the majority of those with guidelines, however, incorporate neither general stewardship principles (19/31, 61%) nor the AWaRe categorization (20/31, 65%). The WHO AWaRe categorization of antibiotics is an initiative to facilitate AMS, which takes into consideration the potential of different antibiotic classes to cause resistance when used for the treatment of clinical infections. It is used as an early detection tool for inappropriate antibiotic use and signals the need for intervention (World Health Organization, 2015).

Appropriate agencies of the Ministry of Health need to rise up to the responsibility of providing direction to the development of context-specific antibiotic treatment guidelines with technical support and training from relevant stakeholders and partners, including the WHO. Antimicrobial treatment of infection syndromes using up-to-date clinical guidelines improves the quality of decision-making, more so in resource-limited countries with low human and diagnostic capacities. Studies have shown that the availability of antimicrobial treatment guidelines significantly increased the odds of receiving appropriate treatments (OR = 6.44, 95% CI = 4.81–8.63) and reduced the relative risk (RR) of mortality (RR = 0.65, 95% CI = 0.54–0.80, *p* < 0.0001) (Schuts et al., 2016; Maina et al., 2020). A survey of 14 Kenyan county hospitals revealed a reduced prevalence of inappropriate antibiotic use in pediatric medical and neonatal units where guidelines are available as compared with adult medical units where guidelines are absent (Maina et al., 2020). Hospital-based or regional guidelines are common in high-income countries, whereas national guidelines are relied upon for the management of cases in LMICs (Maina et al., 2021).

The issue of poor guideline utilization is caused not just by physical unavailability but also by lack of adherence. Surveys in South Africa and Nigeria corroborated the poor guideline compliance and reported that only 45% and 39%, respectively, of prescriptions, were dispensed in agreement with local or national antimicrobial guidelines (Gasson et al., 2018; Aboderin et al., 2021). Dhingra et al., in their review of several studies, also reported antibiotic use not complying with guidelines, causing more drug-resistant infections and increased healthcare costs (Dhingra et al., 2020). A report on the situation analysis of AMU in Ghana also documented the poor adherence to existing guidelines in the management of cases (Yevutsey et al., 2017). The prescription of antibiotics in countries is influenced by personal preferences and the experiences of prescribers rather than by the treatment guidelines (Porter et al., 2021).

Countries should harness human resources and expertise across relevant spheres, with coordination from the central governmental agency, to develop clinical guidelines utilizing local country-wide cumulative data, taking advantage of global surveillance initiatives such as GLASS (Global Antimicrobial Resistance and Use Surveillance System) and recent laboratory infrastructure development efforts by foreign partners. In addition, deploying simple technological innovations has shown great potential for improving compliance with and utilization of clinical guidelines. A pilot study in four African countries showed that mobile applications can provide a simplified way of promoting the appropriate use of antimicrobial drugs, aligning such with suitable guidelines (Olaoye et al., 2020).

### AMS education and training

Despite education and training being the cornerstone of a successful AMS program, our findings show that the level of AMS education is low among healthcare workers and in schools at the basic, primary, and secondary levels. Education is a proven tool for enshrining the AMS principles in low-resource settings, with a significant impact on the prescribing of antimicrobials (Oshun et al., 2021). A systematic review of African studies highlighted the effectiveness of education and training in promoting the appropriate use of antibiotics for case management (Akpana et al., 2020). The efficacy of education and training was further elucidated in an interventional project carried out in Uganda, which included 86 health practitioners (HPs) and 227 community health workers (CHWs) in training workshops and over 300 primary school pupils in sensitization on IPC, AMR, and AMS. The training enhanced practices in the majority of HPs (92.2%) and CHWs (90.3%), including improved hand washing (in 57.3% and 81%, respectively), increased use of clinical guidelines in 52.9% of HPs, reduced quantities of unnecessary antibiotic prescribing in 51.1% of HPs, increased use of treatment guidelines for childhood illnesses in 39.8% of CHWs, and increased drive for stewardship advocacy among CHWs (Musoke et al., 2020).

A deficiency of knowledge on AMS is conspicuous within Africa (Rogers Van Katwyk et al., 2018). Even in countries where there is relatively more stewardship effort, continuous education is still rudimentary, especially at the basic healthcare level (Engler et al., 2021). A review of AMS training in medical education, which included 25 studies involving undergraduate medical trainees, showed a substantial knowledge gap in appropriate AMU, with those with formal training likely to be better equipped for future practice (Rogers Van Katwyk et al., 2018). The teaching of AMS principles should be a priority in primary, secondary, and undergraduate education, as education is pivotal within the framework of “one health” to raising well-informed adults that will show concern for the burden of AMR and be part of the solution at every opportunity (Marvasi et al., 2021). Several guidelines recommend robust strategies for educating physician assistants, nurse practitioners, and medical, pharmacy, and nursing students and trainees on the basic AMS principles. This is well suited for Africa to develop a curriculum with multidisciplinary inputs and strong political support, taking advantage of the available templates, including the WHO curriculum, for the education and training of health workers (World Health Organization, 2019). This should be done with strong consideration of multidisciplinary inputs and political support for successful implementation (Gyssens, 2018).

### Diagnostic stewardship

A functional clinical bacteriology laboratory is pivotal to the successful case management of infections and AMS (Jacobs et al., 2019). The WHO Global Action Strategy for AMR containment prioritizes laboratory-based surveillance as one of the key strategies for containing antibiotic resistance (WHO, 2001); laboratories are, however, the most neglected and least funded in the healthcare system (Ndihokubwayo et al., 2013). This study reveals that about 30% of countries in the region still lacked a coordinated country structure and mechanism for pathogen identification and susceptibility testing, and this finding corroborates the low laboratory diagnostic capacity in Africa due primarily to the lack of modern equipment and essential laboratory consumables (Petti et al., 2006). This has presented challenges for routine testing and reporting, with adverse consequences on patient care and limited AMR surveillance locally and nationally (World Health Organization, 2021). This was well highlighted in a report published by the WHO and partners, revealing weaknesses in national public health laboratories in several African countries (Centers for Disease Control and Prevention, 2009). However, there is a call for renewed efforts to strengthen the laboratory system in the WHO AFRO, and each country should focus on developing a national laboratory policy and strategic plan, set up a national reference laboratory, and streamline its funding to existing ministry and parastatal budgets, and put mechanisms in place for the effective maintenance of laboratory equipment and distribution of supplies (WHO, 2008).

Despite low country-level efforts at upgrading laboratories in LMICs, there have been several initiatives to improve the laboratory capacity in the region. Among these supports for Africa and Asia, the Fleming Fund Country Grant is noteworthy, which supports up to 24 countries to tackle AMR, incorporating the strengthening of laboratory capacity and other interventions (Fleming Fund, 2018). However, to ensure quality-assured laboratory results and good quality surveillance data, more state commitments and non-governmental support are still needed for sustained efforts and a wider reach for a more secure laboratory service with better human and infrastructural resources, as well as regular top-quality reagents and consumables.

## Conclusions

There are wide gaps in the optimization of the use of antimicrobials across countries in the WHO African region, with only 1 out of 31 countries being at the advanced level of implementation of the national core elements for the improvement of AMU. Government efforts face challenges of poor funding, lack of policies and plans, and inadequate governance structure. Moreover, there is low country-level utilization of the WHO AWaRe classification as a stewardship tool integrated into the national EML and the clinical guidelines for patient management. The existing regulations to ensure prescription-only sales of antimicrobials are not effective due to the lack of enforcement. Training of healthcare professionals and a pre-service curriculum incorporating AMR are not in existence in many countries. Surveillance of AMC to inform improvements in AMU is uncommon, with no structure in place in these countries. Gaps lay bare the necessity for a more visible political and government leadership that will promote the responsible use of antimicrobials across societal sectors within the region of Africa.

## Data availability statement

The raw data supporting the conclusions of this article will be made available by the authors, without undue reservation.

## Author contributions

WF and AOA conceived and designed the work. WF, AOA, AY, ATA, LG, OK, OH, and OB contributed to the acquisition, analysis, and interpretation of data. WF and AOA drafted the manuscript. All authors provided critical revisions for important intellectual content. All authors contributed to the article and approved the submitted version.

## Funding

The UK Fleming Fund and Germany BMG supported data collection using the WHO AMS assessment tools across the member states in the WHO African region.

## Acknowledgments

The authors thank all focal persons in all WHO AFRO countries and WHO country offices that provided and facilitated responses to the antimicrobial stewardship assessment tool.

## Conflict of interest

The authors declare that the research was conducted in the absence of any commercial or financial relationships that could be construed as a potential conflict of interest.

## Publisher’s note

All claims expressed in this article are solely those of the authors and do not necessarily represent those of their affiliated organizations, or those of the publisher, the editors and the reviewers. Any product that may be evaluated in this article, or claim that may be made by its manufacturer, is not guaranteed or endorsed by the publisher.
